# Primary T-cell Vitreoretinal Non-Hodgkin Lymphoma: A Case Report and Literature Review

**DOI:** 10.7759/cureus.41341

**Published:** 2023-07-04

**Authors:** Andrew Low, Rhuen Chiou Chow, Ang Ee Ling, Nurliza Khaliddin

**Affiliations:** 1 Department of Ophthalmology, University Malaya Medical Centre, Kuala Lumpur, MYS; 2 Department of Ophthalmology, Hospital Pulau Pinang, Ministry of Health Malaysia, George Town, MYS

**Keywords:** pvrl, intravitreal methotrexate, choroidal biopsy, non-hodgkin lymphoma, primary intraocular lymphoma, t-cell lymphoma, primary vitreoretinal lymphoma

## Abstract

A 72-year-old Chinese male presented with unilateral left eye panuveitis, then diagnosed as bilateral T-cell primary vitreoretinal lymphoma (T-PVRL) through chorioretinal biopsy and immunohistochemistry. No CNS nor systemic involvement was found at diagnosis. Despite initiating intravenous and intrathecal chemotherapy and intravitreal methotrexate, the disease eventually spread to the fellow eye with subsequent recurrence and systemic metastasis. To our knowledge, no cases of T-PVRL treated in a silicone-filled eye were reported in the literature. T- PVRL is exceedingly rare, with most PVRL being the malignant B-cell variant. This case highlights the challenges encountered throughout the treatment course of this aggressive entity, including the administration of intravitreal methotrexate in a silicone oil-filled eye. The poor overall survival rate and grim prognosis of T-PVRL are highlighted. Therefore, we recommend prompt tissue biopsy and immediate initiation of systemic chemotherapy and intravitreal methotrexate.

## Introduction

Primary vitreoretinal lymphomas (PVRL), previously known as primary intraocular lymphomas, are rare but the most prevalent type of intraocular lymphoma. PVRL is a highly aggressive high-grade non-Hodgkin lymphoma strongly linked to primary CNS lymphoma (PCNSL), with approximately 80% of PVRL cases eventually developing PCNSL, whereas 20% of PCNSL cases present with PVRL. The estimated annual incidence of PVRL is 0.46 per 100,000 persons [1]. Most PVRLs are classified as diffuse large B-cell lymphomas, according to the current WHO classification for lymphomas [2], whereas primary intraocular T-cell lymphomas are exceedingly rare. Intraocular lymphomas mainly affect elderly patients and can be unilateral (20%) or bilateral (80%). The vitreoretinal variety typically is not associated with systemic involvement; however, it may sometimes affect the brain before or after ocular manifestations. PVRL remains a diagnostic and therapeutic challenge due to the lack of effective therapeutic tools and delays in diagnosis, which may result in poor prognosis. Herein, we report a case of a patient with primary T-cell vitreoretinal non-Hodgkin lymphoma diagnosed via a chorioretinal biopsy.

## Case presentation

**Figure 1:**
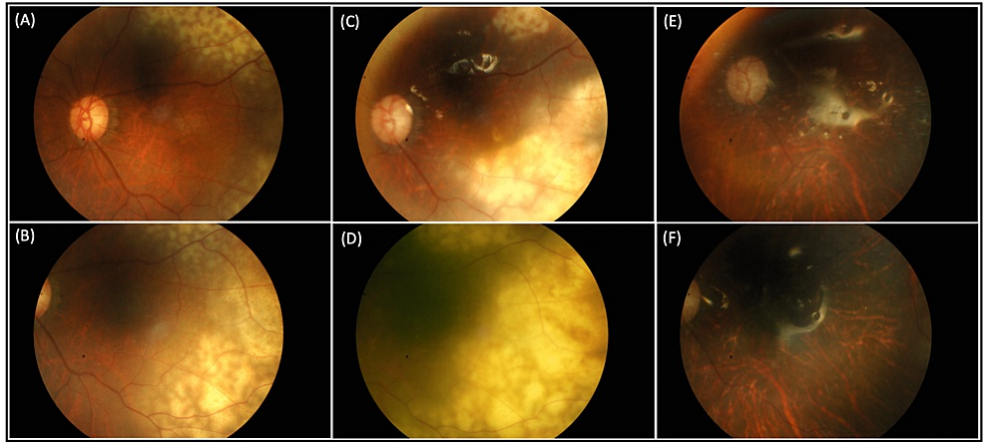
Fundus photo progression of the posterior pole (A, B) The fundus photo of the left eye shows multiple confluent deep chorioretinal lesions at the superotemporal and inferotemporal periphery retina at the initial presentation. (C, D) Progression of the lesions to more periphery retina and posterior pole. (E, F) Resolution of the lesions with scar formation after completion of systemic chemotherapy and 15 doses of intravitreal methotrexate 0.2 mg

A 72-year-old Chinese man with a history of ischemic heart disease, normal-tension glaucoma, and previous left eye vitrectomy for rhegmatogenous retinal detachment presented with unilateral panuveitis in the left eye. Examination revealed 2+ anterior chamber cells and multiple confluent deep chorioretinal lesions in the superotemporal (Figure 1A) and inferotemporal mid-periphery retina (Figure 1B).

**Figure 2:**
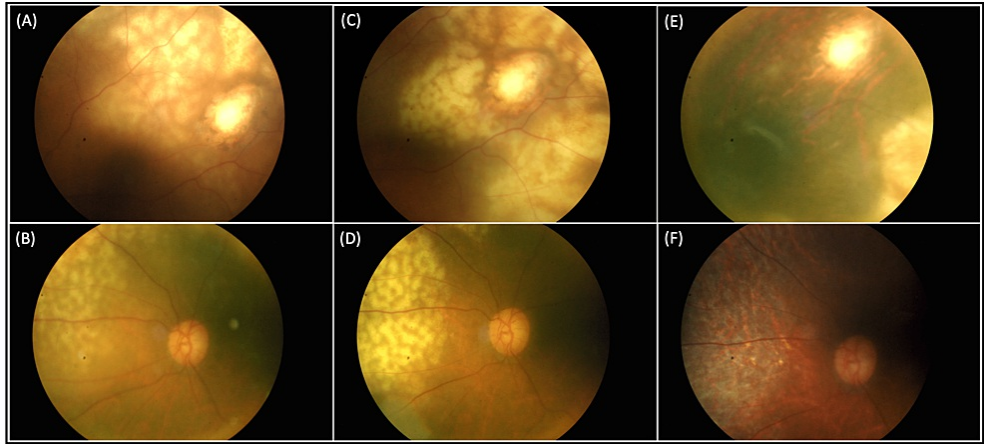
Fundus photo progression of the periphery retina (A, B) Fundus photo of the left eye showing multiple chorioretinal lesions spreading around the previous retinectomy scar and superonasal periphery retina. (C, D) Progression of lesions with centripetal extension towards the posterior pole. (E, F) Resolution of the lesions with scar formation after treatment

There were no vasculitis or vitritis present. The visual acuity was 6/9 OD, 6/12 OS. Optical coherence tomography of the left macula showed minimal subretinal fluid with multiple lobulated choroidal elevations. Differential diagnoses included ocular tuberculosis, masquerade syndromes, syphilis, and sarcoidosis. Further investigations, including serum QuantiFERON-tuberculosis, toxoplasmosis, cytomegalovirus, HIV, venereal disease research laboratory test, and hepatitis B and C, were negative. Fluorescein angiography showed staining of the choroidal lesions. Late dye pooling was seen temporally at the area of lesions. Otherwise, there was no vasculitis, disc leakage, or capillary fallout. MRI orbits showed left eye choroidal thickening. There were no CNS lesions on brain sections. Within the following three months, the choroidal lesions resembling leopard spots increased to involve 360 degrees of periphery arcades (Figure 2B) and eventually the posterior pole (Figure 1C).

**Figure 3:**
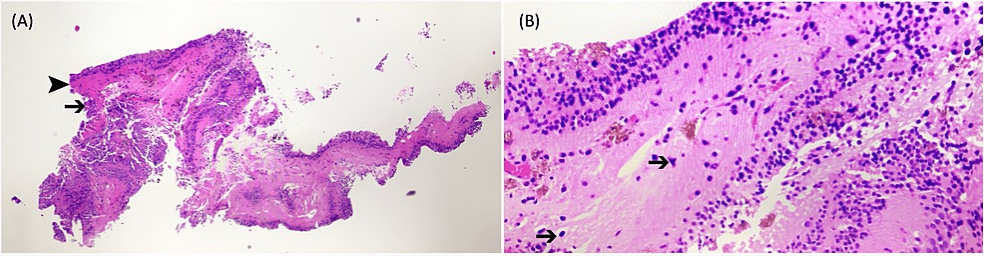
Histopathology slides of the chorioretinal lesion biopsy (A) Cell block of the chorioretinal biopsy specimen with hematoxylin and eosin stain showing levels of the retina (➤) and choroid (➔). The retina is composed of a benign multilayered epithelium with brownish pigment. (B) The underlying fibrovascular stroma of the choroid is infiltrated by singly dispersed clumps and loose sheets of medium to large atypical Ivmphoid cells (➔) with ill-defined cytoplasm. These atypical cells display irregular nuclear contour, hyperchromatism, and mitotic activity. No tumor necrosis is present. A few of the atypical cells involve the retina

There was accompanying grade one vitritis. As his eye was previously vitrectomized, we proceeded with a vitreous tap which yielded negative culture and cytology results. In light of this, we proceeded with left pars plana chorioretinal biopsy and silicone oil 1300 cs tamponade. The chorioretinal histopathology examination returned positive for high-grade T-cell non-Hodgkin lymphoma (Figure 3).

**Figure 4:**
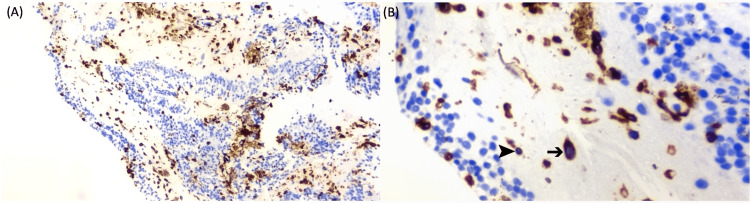
Immunohistochemistry slides of the chorioretinal lesion biopsy (A) Immunohistochemistry staining of the cell block. (B) Atypical lymphoid cells (➔) express CD3, CD8, CD30, and CD56. Ki-67 proliferative index is about 80%. These cells stain negative for ALK, bcl-6, CD4, CD5, CD7, CD10, CO20, EMA, and PAX5. Normal lymphoid cells (➤)

Immunohistochemistry showed that atypical lymphoid cells express CD3, CD8, CD30, and CD56. Ki-67 proliferative index is about 80%. These cells stain negative for ALK, bcl-6, CD4, CD5, CD7, CD10, CD20, EMA, and PAX5 (Figure 4).

He was referred to a hematologist for co-management and treatment. A thorough search for systemic involvement, including a staging contrast-enhanced computerized tomography of the thorax, abdomen, and pelvis, bone marrow aspirate and trephine biopsy, and CSF cytology were negative. The diagnosis of PVRL was made, and treatment was commenced promptly. The patient was started on a chemotherapy regimen of cyclophosphamide, epirubicin, vincristine, prednisolone, etoposide, and intrathecal methotrexate. He received concurrent left eye intravitreal methotrexate biweekly in the initial month, which was gradually tapered in frequency over 12 months. A half dose was used (0.2 mg) as this was an oil-filled eye. His vision was 6/18 OD and CF OS (Figure 1E) at the end of six cycles of systemic chemotherapy. The choroidal lesions in the left eye were indolent (Figure 1F). A full-body PET scan showed no active lesions. However, three weeks after completing systemic chemotherapy, the patient complained of decreased vision and floaters in his right eye. His visual acuity worsened to 6/24 OD. There were occasional right eye anterior chamber cells and new leopard spot lesions in the far peripheral retina. There were dense vitritis which partially obscured the fundus view. A vitreous tap showed atypical CD3-positive lymphoid cells, suggesting T-cell lymphoma. Systemic chemotherapy was deferred as there was no systemic involvement based on the PET scan. Hence, we proceeded with intravitreal methotrexate 0.4 mg (full dose) for his right eye. Regression of the left eye choroidal lesions was seen after 12 doses of intravitreal methotrexate; thus, the treatment was stopped. However, two months later, disease recurrence was seen in the left eye necessitating intravitreal methotrexate reloading. Unfortunately, a repeat PET scan three months after chemotherapy completion revealed metastasis to various locations, including bones, bilateral inguinal lymph nodes, and lungs. The hematology team recommended salvage chemotherapy, but the patient experienced an acute myocardial infarction upon admission for initiation of chemotherapy. His condition deteriorated, and he developed sepsis due to his immunocompromised state. He was given supportive treatment, multiple platelets, and blood transfusions for pancytopenia, but he eventually succumbed to the disease after two months.

## Discussion

The therapeutic challenges associated with PVRL are twofold, encompassing frequent local and CNS relapses. According to various studies, approximately 56% to 90% of PVRL patients eventually experience CNS dissemination within 30 months [3]. The median progression-free survival is typically between 18 and 29 months, whereas overall survival ranges from 58 to 75 months [4]. Regrettably, despite receiving systemic and local treatments, our patient succumbed to metastasis after battling the disease for 16 months. A comprehensive review of the available literature showed that several local and systemic therapies are available for PVRL, but the optimal treatment has yet to be defined. The crucial point in determining the therapeutic decision is CNS involvement and whether the vitreoretinal lymphoma is limited to the ocular. The treatment goals in PVRL without PCNSL are controlling intraocular disease and preventing CNS dissemination. If unilateral involvement is observed, local therapy should be considered, and it has been demonstrated that there is no significant difference in the recurrence rate when standard local therapy or systemic therapy is used. Whether to administer systemic treatment to patients with isolated PVRL remains a subject of ongoing debate, and the identification of definitive predictive factors for CNS dissemination has yet to be established [2].

Intravitreal chemotherapy is an effective method to achieve rapid intraocular cytotoxic drug concentration. In PVRL, variable doses and regimens of methotrexate and rituximab injections have been proposed. Although data on the ocular bioavailability of intravitreal chemotherapy are limited, studies have shown that a single intravitreal injection of 0.4 mg of methotrexate remains effective for more than five days [5]. Intravitreal methotrexate performed under topical anesthesia is a safe and effective treatment for intraocular and retinal diseases. The most common therapeutic regimen consists of intravitreal methotrexate administered at a dose of 0.4 mg in 0.1 mL twice a week for four weeks (induction phase), then once a week for eight weeks (consolidation phase), then once a month for nine months (maintenance phase), for a total of 25 injections. With this treatment regimen, recurrences are infrequent, and only a few complications have been described, like corneal epitheliopathy and transient rise in intraocular pressure [6]. Another treatment regimen consists of intravitreal methotrexate twice weekly for four weeks as an induction phase, followed by a predetermined number of injections over one year or guided by clinical response or IL-10 levels in the aqueous humor.

Our patient's treatment plan involved a scheduled regimen of twice-weekly intravitreal methotrexate for one month, followed by weekly intravitreal methotrexate for another month. Subsequently, biweekly intravitreal methotrexate for one month and monthly injections for the next nine months, resulting in a total one-year duration of treatment. However, due to the presence of silicone oil in the left eye, a half-dose of methotrexate (0.2 mg/0.1 mL) was administered in that eye, while the fellow eye received a full dose of methotrexate (0.4 mg/0.1 mL). It was postulated that the clearance of methotrexate might be delayed when the vitreous is replaced by silicone oil. Due to toxicity concerns, Hardwig et al. reduced the dosing from the standard 0.4 mg to 0.2 mg in a vitrectomized, silicone-filled eye [3]. However, analogous pharmacokinetic studies have not been conducted in silicone-filled eyes. One primary concern is that the clearance of intravitreal methotrexate might be delayed in vitrectomized eyes filled with silicone oil, which could lead to a higher effective cumulative dose of intraocular methotrexate. In turn, this could cause toxicity issues in the patient. Another concern is that methotrexate may be less soluble in silicone oil than the residual fluid at the retinal interface, which could result in a higher effective concentration of methotrexate at the retinal interface. Consequently, intravitreal methotrexate in a silicone-filled eye should be approached with a heightened concern for potential toxicity [6].

Zhou et al. proposed a modified approach for intravitreal methotrexate injections, wherein they transitioned directly from the induction phase to the maintenance phase. This modified protocol resulted in a reduced frequency of injections while maintaining therapeutic effectiveness. Importantly, they observed a lower incidence of corneal epitheliopathy associated with this approach [7]. In a separate study, Giuffrè et al. utilized a combined intravitreal treatment strategy involving alternating administrations of methotrexate and rituximab. Their treatment regimen consisted of four weeks of alternating injections and biweekly administrations for three months. Notably, this treatment approach demonstrated favorable clinical outcomes [8]. The main adverse effects of intravitreal methotrexate are transiently raised intraocular pressure, epithelial keratopathy, and drug resistance, with rare vision-threatening complications such as intraocular hemorrhage, retinal detachment, and endophthalmitis. Another alternative approach involves the intravitreal administration of rituximab at a dosage of 1 mg/0.1 mL for four weeks, with subsequent course repetition depending on the patient's clinical response. Cicinelli et al. conducted a study wherein they observed that 44% of patients achieved a complete disappearance of PVRL following three rituximab injections, while 56% exhibited partial or no remission [9].

External-beam ocular radiotherapy (EBRT) is an alternative local treatment option that provides effective disease control but does not offer protection against CNS relapse [8]. EBRT is a favorable choice for patients with bilateral disease. Notably, the risk of cataracts and radiation retinopathy can be minimized by utilizing lower doses of EBRT, typically below 30 Gy [4]. De la Fuente et al. implemented a treatment approach for PVRL patients involving bilateral radiation therapy followed by systemic methotrexate [10]. Their study findings demonstrated a lower incidence of CNS spread (37.5%) with a median follow-up of 68 months, compared to other studies reporting rates ranging from 56% to 85% [3, 11]. The rationale behind this management strategy is rooted in the possibility that certain PVRL patients may have undetectable occult CNS involvement, which cannot be effectively addressed solely through local therapies. There were no comparative studies that exist to determine the preferred first-line treatment between radiotherapy and intravitreal chemotherapy for PVRL. Hence, the choice should consider disease laterality, patient preference, and practical factors.

The International PCNSL Collaborative Group suggested high doses of systemic chemotherapy, intravitreal chemotherapy, and/or ocular radiotherapy, even in the absence of PCNSL [3]. Empirical systemic treatments for ocular lymphomas typically rely on high-dose methotrexate regimens based on the anatomical and functional similarities between the blood-brain barrier and the blood-retinal barrier [12]. However, more knowledge is needed regarding the pharmacokinetics of drugs administered systemically in ocular tissues and fluid, as only a few studies on a small number of patients have explored this topic. For instance, IV high-dose methotrexate and aracytine were found in the aqueous and vitreous humor shortly after injection [13]. Despite systemic high-dose methotrexate chemotherapy as a first-line treatment, there is a lack of consensus on whether combining it with local treatment decreases the risk of CNS relapse. In a retrospective study by Hashida et al., a combination of local and systemic HD methotrexate treatments significantly delayed CNS relapse in patients [14].

Systemic therapy for PVRL consists of two stages: induction and consolidation. The induction treatment primarily involves high-dose methotrexate as a standalone therapy or in combination with other agents. Multiple studies have demonstrated favorable response rates to methotrexate in PVRL cases with CNS or systemic involvement. When used as a monotherapy, remission rates of up to 72% have been reported, while combination therapies have shown remission rates of up to 94-100% [15]. Following the findings from the International Extranodal Lymphoma Study Group (IELSG) 32 trial, the MATRix combination therapy comprising methotrexate, cytarabine, thiotepa, and rituximab has emerged as the new standard chemotherapy for patients under the age of 70 as a first-line treatment for PCNSL. The group treated with the MATRix regimen demonstrated a complete remission rate of approximately 50% at 30 months. In comparison, the group treated with methotrexate and cytarabine alone had a remission rate of only 23%, while the group receiving methotrexate, cytarabine, and rituximab had a remission rate of 30% [16].

Additionally, a small prospective series of 11 patients treated by Kaburaki et al. with a conventional PCNSL-like treatment consisting of systemic IV rituximab, high-dose methotrexate, procarbazine, and vincristine plus reduced-dose whole-brain radiotherapy (23.4 Gy) and IV high-dose cytarabine combined with intravitreal methotrexate reported encouraging results. After a median follow-up of 40 months, the estimated four-year cumulative incidence of CNS progression was only 10% [17]. After high-dose methotrexate induction therapy, 60% of patients usually respond completely. In the event of a relapse, intravitreal chemotherapy and EBRT can be administered as a standalone treatment. The use of immunochemotherapy in conjunction with an autologous stem cell transplant (ASCT) has been used in patients with relapsed or refractory PVRL as a means to overcome the BRB [18]. Due to the favorable CNS bioavailability of thiotepa and busulfan, the immunochemotherapy regimen primarily involved high-dose thiotepa, busulfan, and cyclophosphamide. The feasibility and efficacy were also demonstrated in patients with poor-prognosis systemic lymphoma, including those with CNS involvement [19]. Retrospective and prospective studies by Soussain et al. included patients with relapsed or refractory PVRL and PCNSL using immunochemotherapy plus ASCT regimen [18,20]. These studies confirmed the effectiveness of this treatment in patients with relapsed or refractory PVRL and PCNSL, with no significant differences in outcomes between the two groups. Despite the limited sample size of patients with PVRL due to the rarity of this disease and the absence of comparative studies, immunochemotherapy plus ASCT remains a viable treatment option for relapsed PVRL in younger patients without severe comorbidities.

In summary, for PVRL patients with unilateral involvement, local intravitreal therapy should be considered. If local recurrence occurs without bilateral eye or CNS involvement, either resumption of local intravitreal therapy or local radiation (30 Gy) can be given. If there is bilateral vitreoretinal recurrence with or without CNS involvement, treatment with ASCT can be provided in conjunction with local therapy. In the case of initial bilateral ocular involvement with PVRL, systemic methotrexate, rituximab, and temozolomide may be used with local intravitreal treatment with rituximab and methotrexate.

## Conclusions

PVRL is elusive and challenging to diagnose, often leading to treatment delays impacting patient survival. The diagnosis can be difficult as, in our case, the initial vitreous biopsy yielded negative results; hence, we proceeded with an immediate diagnostic vitrectomy and chorioretinal biopsy, which confirmed the diagnosis. Early recognition of PVRL and initiating systemic chemotherapy and intravitreal methotrexate can be life-saving. However, more in-depth research needs to be focused on the safety and efficacy of half-dose intravitreal methotrexate injection in silicone-filled eyes and the detection of gene mutation in aqueous and vitreous samples, which is still lacking in the literature.
